# Caesarean section, but not induction of labour, is associated with major changes in cord blood metabolome

**DOI:** 10.1038/s41598-019-53810-1

**Published:** 2019-11-26

**Authors:** Linda Marchioro, Engy Shokry, Aisling A. Geraghty, Eileen C. O’Brien, Olaf Uhl, Berthold Koletzko, Fionnuala M. McAuliffe

**Affiliations:** 1 Division of Metabolic and Nutritional Medicine, Department of Paediatrics, Dr. von Hauner Children’s Hospital, University Hospital, LMU Munich, Munich, Germany; 2 UCD Perinatal Research Centre, Obstetrics and Gynaecology, School of Medicine, University College Dublin, National Maternity Hospital, Dublin, Ireland

**Keywords:** Metabolomics, Biochemical networks

## Abstract

The physiology of how prelabour caesarean section (PCS) and induction of labour (IOL) in comparison to spontaneous vaginal delivery (SVD) has not been fully clarified yet. We measured 201 cord blood (CB) phospholipids and energy metabolites via LC/MS-MS in 109 newborns from the ROLO Kids study; metabolites were compared across the three parturition groups via linear mixed models with correction for multiple testing. In comparison to SVD, PCS babies had lower non-esterified fatty acids (NEFA), including sum of NEFA (p < 0.001), and trends for lower acylcarnitines. The lack of hormonal stimuli, especially catecholamines and cortisol, may underlie the metabolic changes involving gluconeogenesis from fatty acid oxidation (FAO) in PCS born infants. IOL and SVD infants showed no significant differences in metabolites, but ratios estimating carnitine palmitoyltrasferase 1 activity (precursor for FAO) were slightly higher in IOL than in SVD. Thus, IOL does not induce metabolic disadvantage when compared to SVD, though post-natal gluconeogenesis might start earlier due to the artificial solicitation in IOL. These data shed light on the physiology of parturition and may contribute to understand how mode of delivery might modulate future metabolic risks.

## Introduction

Caesarean section (CS) and induction of labour (IOL) are surgical and non-surgical approaches to facilitate birth in complicated pregnancies/deliveries or overdue mothers, but their widespread usage is controversial. While the WHO states that “[at] population level, caesarean section rates higher than 10% are not associated with reductions in maternal and newborn mortality rates”^1^, the worldwide rate of CS was estimated in 2016 to be of 18.6%^2^. Birth by CS has been associated, among others, with intestinal microbial colonization^3^ and increased risk for obesity^4^. Pre-labour (elective) CS (PCS) is of particular interest, as it seems to induce physiological changes able to trigger long-term health outcomes^5,6^. IOL, while recommended under specific circumstances^7^, is associated with several obstetrical complications^8^ and often met with adverse maternal perception^9^. The physiological mechanisms of how parturition, and especially IOL, can impact the offspring’s metabolism are largely unclear^6^.

Metabolomics is the study of small molecules which are intermediates or products of metabolic reactions and has found clinical applications in the mechanistic explanation of pathophysiological phenomena. Mode of delivery, however, has received little interest in metabolomics research so far. In 2009, Hyde *et al*. studied the hepatic metabolome of piglets after observing that PCS was associated with higher risk for hepatic steatosis than SVD^10^; four years later, Hashimoto and colleagues used metabolomics to explain the changes in cord blood saccharides associated with caesarean delivery^11^. However, no metabolomics studies have been carried out regarding IOL.

To overcome this paucity of information about how mode of delivery might impact the fetal/neonatal metabolism, we performed a descriptive analysis investigating the association of mode of delivery (SVD, PCS or IOL followed by vaginal delivery) with glucose, c-peptide and 201 metabolites (intermediates of energy metabolism and phospholipids) measured in cord blood. Moreover, we compared infant anthropometry at 6 months, 2, and 5 years across the three mode of delivery groups and compared our population to epidemiological studies.

## Methods

### Subjects

The ROLO Study (Randomised cOntrol trial of LOw glycemic index diet versus no dietary intervention to prevent recurrence of fetal macrosomia), a randomised control trial (RCT) conducted at the National Maternity Hospital, Dublin, Ireland between 2007 and 2012^12^ (Current Controlled Trials ISRCTN54392969), investigated the hypothesis whether an isocaloric low-glycemic index (low-GI) diet in pregnancy could be beneficial in the reduction of birth weight in a population at risk for macrosomia. The study was conducted by randomizing 800 secundigravid mothers with a previous macrosomic child (birthweight >4000 kg) and no underlying metabolic disorders to either receive an educational session about low-GI diet in pregnancy, or standard care only. No difference in the primary outcome, birthweight, was observed^12^. The data here presented have been collected at 6 months (6 m), 2 years (2 y) and 5 years (5 y) within the follow up of the ROLO Study, the ROLO Kids Study. Both studies were carried out in accordance with the Helsinki Declaration of 1975 as revised in 1983. Institutional ethical approval from the National Maternity Hospital was obtained in 2006 for the original ROLO study, and in 2009 for the ROLO Kids 6 months and 2 years follow-up. The ROLO Kids 5 years follow-up was approved by the Ethics (Medical Research) Committee in Our Lady’s Children’s Hospital, Dublin. Informed written maternal consent was obtained during pregnancy and at each subsequent follow-up.

### Anthropometry and clinical data collection

Maternal weight and height, education level, maternal smoking and paternal body mass index (BMI) were collected or measured at the first study visit (median gestational week: 13^th^ week) and maternal BMI was calculated. Gestational weight gain (GWG) was defined as the weight from the last visit pre-partum (38 weeks) minus the weight measured at booking visit. The socio-economic status was assessed by calculating the 2011 Pobal Haase & Pratschke Deprivation Index (HP Index) as previously described^13^.

At birth, information about mode of delivery (spontaneous standard vaginal delivery (SVD), prelabour caesarean section (PCS), induction of labour followed by vaginal delivery (IOL), or emergency caesarean section/other approaches) child sex, weight (kg) and length (cm) were recorded. Systolic and diastolic blood pressure was measured at day 0 and day 1 using a calibrated mobile Trimline aneroid sphygmomanometer (Trimline Medical Products Corp, USA). Infant weight and length/height were additionally measured at the follow-up visits and z-scores for weight (z-weight), length (z-length) and BMI (z-BMI) were calculated using WHO standards^14^. Breast feeding was assessed as any breast feeding reported by parents at the 6 months visit (or 2 years visit, if the information was missing). Information about maternal smoking was collected at 2 years.

### Cord blood collection and clinical parameters

Cord blood was collected at birth and stored at −80 °C until analysis. Glucose, c-peptide, leptin and triglycerides were measured according to previously published methods^15^.

### Metabolomics measurements

136 cord blood samples were measured with a targeted approach at the laboratory of the Division of Metabolic and Nutritional Medicine, von Hauner Children’s Hospital, University of Munich, Germany. Metabolites were measured as previously described^16^ (see also supplemental material for details).

Each batch included 6 quality control (QC) replicates, pooled from a subset of the test samples; batches and analytes with intra-batch coefficient of variation (CV) < 20% (after removal of at most one outlier, defined as QC measurement >2 inter-quartile ranges from next measurement) and inter-batch CV < 30% were included in the analysis. The inclusion of analytes with at most one batch with 20% < intra-batch CV < 30% and/or inter-batch 35% was decided on a case by case basis via boxplots inspection. 185 metabolites passed the quality control. These included: 22 amino acids (AA), 39 non-esterified fatty acids (NEFA), 41 acylcarnitines (AC, included free Carnitine, AC 0), 11 organic acids, 45 phosphatidylcholines (PC: PCaa, with 2 acylester bonds; PCae, with one acylester and one ether bond), 7 lyso-PC (LPC) and 20 sphingomyelins (SM). Additional sums and ratios were computed: five ratios of AC/AC 0, markers for carnitine palmitoyl-trasferase 1 (CPT1), five ratios of AC 2/long chain AC, markers for beta-oxidation, and the sums of NEFA, PCaa, PCae, LPC, total PC and total SM^17^.

### Statistical analysis

All statistical analyses were carried out in R version 3.4.3^18^. To ensure interpretability of the findings, only mothers with uncomplicated SVD, planned PCS or IOL and term babies (>37^th^ gestation week) were included.

### Phenotypic differences

Phenotypic characteristics are presented as median ± interquartile range (IQR), or as n (%). Overall group differences for the phenotypic variables were inspected via Kruskal-Wallis tests, for continuous variables, or chi-square tests, for categorical variables. If p < 0.1, pairwise comparisons (PCS vs. SVD, IOL vs. SVD) were inspected via Wilcoxon test or Fisher’s exact test. For antenatal and perinatal variables, no correction for multiple testing was applied, as we used this information to select which variables should be included in the main models and in the sensitivity analyses. However, we did correct childhood anthropometry measures for multiple testing (Bonferroni correction with m = 23, number of total anthropometry measures from 6 months until 5 years).

### Metabolite differences

Metabolite concentrations were logged; values further away than 4 IQR from the median were defined as outliers and removed. Logged metabolite concentrations were used as dependent variables and mode of delivery (SVD, PCS or IOL) as independent variable in a linear mixed model with random intercept for the batch number (to adjust for batch effects). RCT was not included as model covariate since all groups showed similar proportions of mothers in the intervention and control arms (see results section), and since cord metabolome was not affected by the intervention^16^. The following sensitivity analyses were performed: (1) gestational age (GA), child sex, birth weight and maternal BMI were included one by one as additional covariates; (2) the highest and lowest 2.5% of each metabolite were removed (95% central data); (3) IOL babies born before the 40^th^ gestational week were excluded. The R packages lme4^19^ and lmerTest^20^ were used. P-values were adjusted for multiple testing using Bonferroni correction for m = 201 (number of analytes, sums and ratios). Metabolites associations with p < 0.05/201 ≈ 0.00025 were defined as significant, associations with p < 0.05 as trends. The results for the metabolites models are presented in form of Manhattan plots.

## Results

### Phenotypic characteristics

After removing subjects with missing information about mode of delivery, cases of emergency caesarean section and pre-term babies (<37^th^ gestational week), 109 subjects were included in the analysis. Table 1 presents the participants’ characteristics compared between standard vaginal delivery (SVD, n = 76), planned caesarean section (PCS, n = 13) and induction of labour (IOL, n = 20).

**Table 1:** Characteristics of the studied population. Phenotypic differences are presented as median ± interquartile range (IQR), or as n (%).

	SVD		PCS		IOL		p-values		
	n	Values	n	Values	n	Values	Overall	PCS vs. SVD	IOL vs. SVD
**Maternal and paternal variables**									
RCT group - Intervention	76	32 (42%)	13	6 (46%)	20	13 (65%)	NS	NS	NS
Education - 3rd level at least begun	65	41 (63%)	11	8 (73%)	17	8 (47%)	NS	NS	NS
Maternal age (years)	76	32.95 ± 5.59	13	32.97 ± 1.90	20	32.11 ± 6.35	NS	NS	NS
Maternal early pregnancy BMI (kg/m^2^)	76	26.13 ± 5.33	13	25.22 ± 3.88	19	25.75 ± 5.35	NS	NS	NS
Gestational weight gain (kg)	66	12.10 ± 4.92	11	12.40 ± 2.73	17	13.90 ± 4.40	NS	NS	NS
HP deprivation index score 2011	76	6.95 ± 12.60	13	10.50 ± 10.70	20	8.10 ± 16.18	NS	NS	NS
Mother smoking at baseline - yes	76	2 (3%)	13	0 (0%)	20	1 (5%)	NS	NS	NS
Mother smoking at 2 years - yes	39	3 (8%)	6	1 (17%)	9	3 (33%)	0.066	0.448	0.071
Paternal BMI (kg/m^2^)	35	26.77 ± 4.23	6	30.42 ± 11.82	12	27.84 ± 5.24	NS	NS	NS
**Perinatal variables - anthropometry**									
Child sex - female	76	42 (55%)	13	6 (46%)	20	9 (45%)	NS	NS	NS
Gestational age (days)	76	282 ± 9.00	13	277 ± 8.00	20	288 ± 11.50	0.005	0.015	0.057
Birth length (cm)	67	52.00 ± 3.00	11	53.00 ± 0.75	17	53.70 ± 3.70	NS	NS	NS
Birth weight (g)	76	4070 ± 600.00	13	4005 ± 910.00	20	4105 ± 675.00	NS	NS	NS
Birth waist circumference (cm)	54	33.20 ± 3.08	10	34.00 ± 3.83	15	33.30 ± 2.85	NS	NS	NS
Birth length-for-age (WHO z-score)	67	1.53 ± 1.61	11	1.91 ± 0.48	17	2.44 ± 1.70	NS	NS	NS
Birth weight-for-age (WHO z-score)	76	1.59 ± 1.00	13	1.58 ± 1.35	20	1.54 ± 1.21	NS	NS	NS
Birth weight-for-length (WHO z-score)	67	0.16 ± 1.92	11	0.15 ± 1.16	17	−0.57 ± 2.26	NS	NS	NS
Birth BMI-for-age (WHO z-score)	67	0.76 ± 1.38	11	0.94 ± 1.36	17	0.42 ± 1.17	NS	NS	NS
**Perinatal variables - no anthropometry**									
Cord blood glucose (mmol/l)	67	4.10 ± 1.15	12	2.75 ± 0.62	18	4.45 ± 1.30	< 0.0001	< 0.0001	NS
Cord blood C-peptide (ng/ml)	72	0.41 ± 0.98	13	0.26 ± 0.22	20	0.41 ± 0.51	0.051	0.017	NS
Cord blood leptin (ng/ml)	65	24.02 ± 30.15	11	14.48 ± 7.50	16	27.71 ± 27.11	NS	NS	NS
Cord blood triglycerides (mmol/l)	46	0.47 ± 0.20	8	0.40 ± 0.29	14	0.49 ± 0.32	NS	NS	NS
Baby diastolic BP (day 0) (mmHg)	72	70 ± 9.00	13	71 ± 8.00	19	70 ± 8.50	NS	NS	NS
Baby systolic BP (day 0) (mmHg)	72	120 ± 14.25	13	115 ± 10.00	19	120 ± 14.00	NS	NS	NS
Baby diastolic BP (day 1) (mmHg)	70	70 ± 12.25	13	68 ± 10.00	20	68 ± 12.50	NS	NS	NS
Baby systolic BP (day 1) (mmHg)	70	110.50 ± 12.25	13	110 ± 18.00	20	115 ± 12.75	NS	NS	NS
Any breastfeeding - Yes	40	32 (80%)	6	3 (50%)	10	6 (60%)	NS	NS	NS
**6 months anthropometry**									
Child sex - female	47	26 (55%)	9	4 (44%)	11	4 (36%)	NS	NS	NS
Age at 6 m visit (years)	43	0.52 ± 0.08	9	0.51 ± 0.06	10	0.53 ± 0.05	NS	NS	NS
6 m length* (cm)	47	69.50 ± 3.50	9	69.50 ± 2.50	11	70.00 ± 1.00	NS	NS	NS
6 m weight* (kg)	47	8.37 ± 1.05	9	8.17 ± 1.21	11	8.60 ± 0.97	NS	NS	NS
6 m BMI* (kg/m^2^)	47	17.26 ± 1.57	9	16.91 ± 1.64	11	18.11 ± 1.32	NS	NS	NS
6 m waist circumference* (cm)	47	44.10 ± 4.20	9	44.00 ± 3.90	11	44.00 ± 2.10	NS	NS	NS
6 m length-for-age* (WHO z-score)	43	0.93 ± 1.18	9	1.41 ± 0.44	10	1.37 ± 0.36	NS	NS	NS
6 m weight-for-age* (WHO z-score)	43	0.75 ± 1.07	9	0.52 ± 0.81	10	1.24 ± 0.96	NS	NS	NS
6 m weight-for-length* (WHO z-score)	47	0.22 ± 1.12	9	−0.05 ± 0.82	11	0.75 ± 0.86	NS	NS	NS
6 m BMI-for-age* (WHO z-score)	43	−0.03 ± 1.08	9	−0.17 ± 0.83	10	0.71 ± 0.78	NS	NS	NS
**2 years anthropometry**									
Child sex - female	48	26 (54%)	8	4 (50%)	14	7 (50%)	NS	NS	NS
Age at 2 y visit (years)	48	2.03 ± 0.08	8	2.02 ± 0.07	10	2.04 ± 0.10	NS	NS	NS
2 y length* (cm)	47	89.00 ± 4.25	8	92.75 ± 4.88	14	89.00 ± 4.25	NS	NS	NS
2 y weight* (kg)	47	12.40 ± 1.95	8	14.45 ± 1.25	14	13.00 ± 1.90	NS	NS	NS
2 y BMI* (kg/m^2^)	47	15.67 ± 2.05	8	16.62 ± 0.77	14	15.99 ± 1.34	NS	NS	NS
2 y waist circumference* (cm)	46	49.95 ± 5.75	8	51.95 ± 3.90	14	51.90 ± 3.48	NS	NS	NS
2 y length-for-age* (WHO z-score)	47	0.84 ± 1.28	8	1.80 ± 1.21	10	0.45 ± 1.43	NS	NS	NS
2 y weight-for-age* (WHO z-score)	47	0.30 ± 0.99	8	1.49 ± 0.64	10	0.43 ± 1.23	NS	NS	NS
2 y weight-for-length* (WHO z-score)	47	0.00 ± 1.52	8	0.62 ± 0.41	14	0.06 ± 0.95	NS	NS	NS
2 y BMI-for-age* (WHO z-score)	47	0.00 ± 1.64	8	0.57 ± 0.42	10	−0.32 ± 0.74	NS	NS	NS
**5 years anthropometry**									
Child sex - female	53	32 (60%)	12	6 (50%)	14	5 (36%)	NS	NS	NS
Age at 5 y visit (years)	53	5.13 ± 0.19	12	5.14 ± 0.09	14	5.14 ± 0.19	NS	NS	NS
5 y length* (cm)	51	112.70 ± 6.15	11	112.80 ± 4.40	13	112.30 ± 3.30	NS	NS	NS
5 y weight* (kg)	51	19.60 ± 3.40	11	21.20 ± 2.30	13	19.00 ± 1.90	NS	NS	NS
5 y BMI* (kg/m^2^)	51	15.84 ± 1.69	11	16.15 ± 1.32	13	15.22 ± 1.62	NS	NS	NS
5 y waist circumference* (cm)	51	53.90 ± 5.60	11	53.20 ± 4.15	13	53.00 ± 4.90	NS	NS	NS
5 y length-for-age* (WHO z-score)	51	0.40 ± 1.35	11	0.41 ± 0.85	13	0.24 ± 0.64	NS	NS	NS
5 y weight-for-age* (WHO z-score)	51	0.34 ± 1.09	11	0.83 ± 0.83	13	0.15 ± 0.57	NS	NS	NS
5 y BMI-for-age* (WHO z-score)	51	0.41 ± 1.07	11	0.66 ± 0.82	13	−0.03 ± 1.23	NS	NS	NS

Overall p-values were calculated via Kruskal-Wallis tests, for continuous variables, or chi-square tests, for categorical variables. If the overall p < 0.1, pairwise comparisons were performed via Wilcoxon test or Fisher’s exact test. P-values for the 23 childhood anthropometry variables were adjusted via Bonferroni method (variables marked with *). Abbreviations: SVD: spontaneous vaginal delivery; PCS: prelabour caesarean section; IOL: induction of labour; NS: non-significant (p ≥ 0.1).

SVD, PCS and IOL groups showed no differences with regards to maternal characteristics or RCT arm.

**Figure 1:**
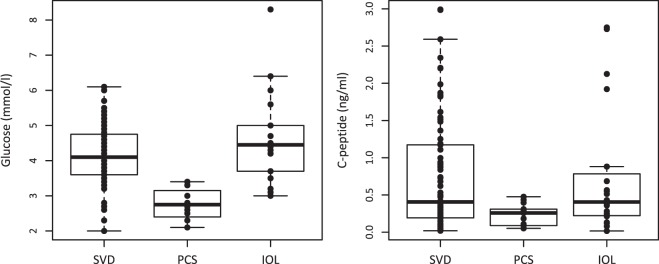
Cord blood concentrations of glucose (left) and C-peptide (right) according to the mode of delivery (SVD: standard vaginal delivery; PCS: prelabour caesarean section; IOL: induction of labour).

At birth, gestational age (GA) and cord blood (CB) glucose showed differences between the three groups (GA: p = 0.005, glucose: p < 0.001). In both cases, PCS was significantly different from SVD, with lower GA (median SVD vs. PCS: 282 vs. 277 days, p = 0.015) and lower glucose (4.10 vs. 2.75 mmol/l, p < 0.001); IOL had a trend for longer GA (median SVD vs. IOL: 282 vs. 288 days, p = 0.057) but no difference in glucose (4.10 vs. 4.45 mmol/l, non-significant (NS)). C-peptide was marginally different in the three groups (p = 0.051) and followed the same pattern as glucose (median SVD vs. PCS: 0.41 vs. 0.26 ng/ml, p = 0.017; IOL: 0.41 ng/ml, difference to SVD: NS). Leptin was not significantly different among the groups, but its median values tended to be lower in PCS than in SVD and IOL babies. Figure 1 shows the boxplot for glucose and C-peptide according to the mode of delivery group.

No differences in birth anthropometry or post-natal blood pressure were identified; the median birthweight was approximately 4000 g in every group (SVD: 4070 g, PCS: 4005 g, IOL: 4105 g).

The rate of breast-feeding initiation tended to be higher in SVD mothers (80%) than IOL (60%) or PCS (50%) (NS) (see Table 1).

No differences between IOL and SVD were observed at any time point, or between SVD and PCS at 6 months and 5 years of age, in infant anthropometry. At 2 years of age, PCS children had higher weight (median SVD vs. PCS: 12.40 vs. 14.45 kg) and weight-for-age z-scores (0.30 vs. 1.49) than SVD, but these differences were not significant after correction for multiple testing.

### Metabolomics analysis

#### Prelabour caesarean section

When compared to SVD, PCS had significantly lower NEFA (29 out of 39 species, as well as the total sum of NEFA). AC 18:2 was significantly lower in PCS, with other AC (AC 10:2, 12:0, 14:0, 14:1, 16:0 Oxo, 16:1) following the same trend. The sensitivity analyses confirmed these results, especially the negative trend for the AC.

Citric and isocitric acid were both lower in the PCS group than in the SVD group. However, due to artefacts in the data, citric acid was only significant in the full model, isocitric acid only in the sensitivity analysis. Trends for lower levels of phenylalanine, isoleucine, valine and ornithine in the PCS group were observed.

AC 4:0 and alpha-aminoadipic acid tended to be higher in PCS than SVD in the main model, but did not pass the sensitivity analysis. The ratios AC 18:0/AC 0 and AC 18:2/AC 0, markers for CPT1 activity, tended to be higher and lower, respectively, in the PCS group; only the latter remained evident also after sensitivity analyses. The remaining markers for CPT1 and beta oxidation were lower in the PCS than in the SVD group, but not significantly.

#### Induction of labour

No significant differences were identified between SVD and IOL, but there were trends for higher CPT1 activity markers (AC 18:0/AC 0, AC 16:0/AC 0, AC 18:1/AC 0) and NEFA 26:0, and a negative trend for free carnitine. NEFA 26:0 and free carnitine did not pass the sensitivity analyses.

**Figure 2:**
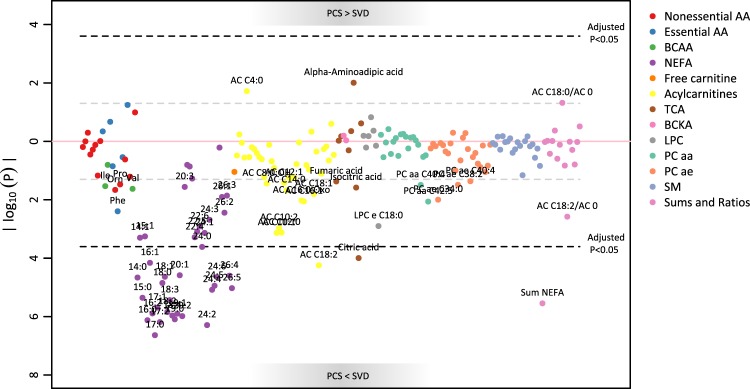
Manhattan plot for the association of metabolites with prelabour caesarean section (PCS) compared to standard vaginal delivery (SVD). Associations were calculated via linear mixed models with random intercept for batch number.

**Figure 3:**
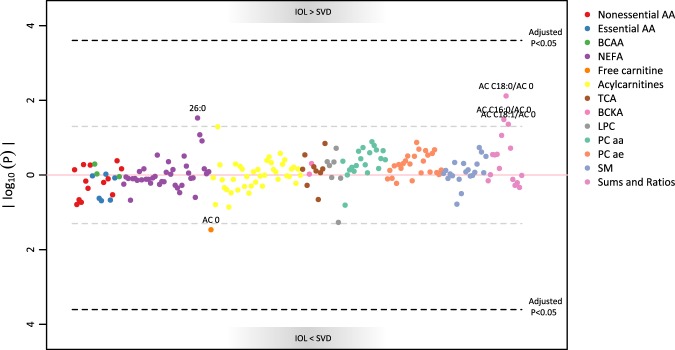
Manhattan plot for the association of metabolites with induction of labour (IOL) compared to standard vaginal delivery (SVD). Associations were calculated via linear mixed models with random intercept for batch number.

Table 2 shows the results for significant metabolites. The results for all metabolites are graphically represented in Manhattan plots (Figs. 2 and 3) and the full table is available as supplemental material.

**Table 2:** Results of the linear models (dependent variable: logged metabolite concentration, independent variable: mode of delivery, random intercept for batch number).

Analyte	Analyte group	n	PCS			IOL		
			Beta	95% Beta CI (Bonferroni)	P-value (Bonferroni)	Beta	95% Beta CI (Bonferroni)	P-value (Bonferroni)
14:0	NEFA	109	−0.745	(−1.370, −0.120)	0.004	−0.080	(−0.603, 0.443)	1.000
15:0	NEFA	109	−0.712	(−1.261, −0.160)	8.86e-04	−0.030	(−0.492, 0.434)	1.000
16:0	NEFA	109	−0.526	(−0.901, −0.153)	1.51e-04	−0.022	(−0.344, 0.290)	1.000
16:1	NEFA	109	−0.619	(−1.177, −0.062)	0.014	−0.031	(−0.498, 0.436)	1.000
16:2	NEFA	109	−0.665	(−1.147, −0.182)	2.59e-04	−0.039	(−0.443, 0.365)	1.000
17:0	NEFA	109	−0.582	(−0.973, −0.189)	4.64e-05	0.022	(−0.305, 0.352)	1.000
17:1	NEFA	109	−0.809	(−1.408, −0.204)	4.09e-04	−0.045	(−0.547, 0.469)	1.000
17:2	NEFA	109	−0.691	(−1.176, −0.205)	1.3e-04	0.045	(−0.362, 0.451)	1.000
18:0	NEFA	107	−0.656	(−1.196, −0.118)	0.003	−0.034	(−0.477, 0.402)	1.000
18:1	NEFA	109	−0.563	(−1.036, −0.089)	0.005	−0.031	(−0.428, 0.365)	1.000
18:2	NEFA	109	−0.744	(−1.287, −0.201)	2.92e-04	−0.063	(−0.518, 0.391)	1.000
18:3	NEFA	109	−1.044	(−1.844, −0.244)	7.36e-04	−0.037	(−0.711, 0.629)	1.000
18:4	NEFA	109	−0.932	(−1.610, −0.260)	2.18e-04	−0.016	(−0.595, 0.543)	1.000
19:0	NEFA	109	−0.510	(−0.873, −0.148)	1.62e-04	0.086	(−0.218, 0.390)	1.000
19:1	NEFA	109	−0.634	(−1.094, −0.170)	2.53e-04	−0.061	(−0.446, 0.336)	1.000
20:1	NEFA	109	−0.493	(−0.910, −0.071)	0.005	−0.041	(−0.390, 0.315)	1.000
20:2	NEFA	109	−0.520	(−0.893, −0.147)	2.08e-04	−0.037	(−0.349, 0.275)	1.000
24:0	NEFA	108	−0.498	(−0.991, −0.008)	0.049	−0.070	(−0.491, 0.342)	1.000
24:2	NEFA	109	−0.499	(−0.846, −0.151)	1.03e-04	0.043	(−0.247, 0.335)	1.000
24:4	NEFA	109	−0.469	(−0.842, −0.096)	0.002	−0.020	(−0.332, 0.293)	1.000
24:5	NEFA	109	−0.393	(−0.711, −0.075)	0.002	−0.015	(−0.281, 0.251)	1.000
24:6	NEFA	108	−0.480	(−0.890, −0.078)	0.005	−0.101	(−0.439, 0.223)	1.000
26:4	NEFA	107	−0.314	(−0.580, −0.049)	0.005	0.006	(−0.217, 0.228)	1.000
26:5	NEFA	107	−0.356	(−0.641, −0.071)	0.002	0.015	(−0.224, 0.254)	1.000
AC 18:2	Acylcarnitines	109	−0.498	(−0.940, −0.055)	0.011	−0.007	(−0.377, 0.364)	1.000
Citric acid	TCA	107	−0.229	(−0.440, −0.018)	0.020	0.019	(−0.158, 0.196)	1.000
Sum NEFA	Sums and Ratios	106	−0.584	(−1.031, −0.146)	5.71e-04	−0.037	(−0.406, 0.317)	1.000

Beta >0 indicates higher concentration of the metabolite in the group PCS or IOL than in SVD (reference group). Only significant associations (Bonferroni-corrected p-value < 0.05) are presented here; for all metabolites see Supplemental Table 1. Abbreviations: PCS: prelabour caesarean section; IOL: induction of labour.

## Discussion

### Metabolism

In this analysis comprising 109 children from the ROLO study, the cord blood of babies born via vaginal delivery, both spontaneously (SVD) or after induction of labour (IOL), showed no differences regarding glucose, c-peptide and leptin concentrations. In the metabolome, no significant differences were identified (Fig. 2), but ratios depicting CPT1 activity tended to be higher in IOL babies. CPT1 contributes to transfer long-chain fatty acids into mitochondria for beta-oxidation^21^. In this context, enhanced perinatal CPT1 activity can be interpreted as preparatory mechanism to postnatal gluconeogenesis, which allows the newborn to transition from the continuous supply of nutrients to intermittent feeding^22,23^. A prerequisite for gluconeogenesis is the availability of substrates such as glycogen^23^, alanine^23^, and ATP from lipids oxidation^24^. We hypothesize that IOL babies, by receiving an artificially dosed hormonal solicitation in the hours before birth, might initiate fatty acid oxidation (FAO) slightly earlier than SVD-born babies, thus the elevated levels of CPT1 activity markers in the cord blood. Despite the small magnitude of this effect, this could become relevant particularly when considering IOL for mothers with metabolic derangements. The WHO guidelines already discourage performing IOL in mothers with (gestational) diabetes with no additional complications, despite recognizing the paucity of data available to this theme^7^.

Prelabour caesarean section (PCS), on the contrary, was associated with profound metabolic changes: most notably, the cord blood of PCS babies had significantly lower levels of glucose and NEFA and trends for lower C-peptide (used here as marker for fetal insulin) and acylcarnitines (AC) than SVD. The differences in glucose and NEFA are already known^24^; on the other hand, the best of our knowledge, the trends for AC have not been reported previously, and only one study observed lower insulin in neonatal sheep born by PCS^25^. During labour, in presence of contractions, a plethora of hormones are released by maternal, fetal and placental tissues to enable the passage of the baby though the vaginal canal and to prepare the baby for the extrauterine life. Two crucial hormones are cortisol and oxytocin. They are secreted both from the mother and the fetus. Fetal cortisol, in particular, is thought to regulate the adrenergic stimulation at birth^22^. Catecholamines have been found responsible for important postnatal adaptation, among others glucose regulation^23,26^. Oxytocin is released discontinuously both by the maternal pituary gland and the fetus to stimulate uterine contractions during labour^27,28^ and has been observed to stimulate the neonate’s insulin and glucagon secretion in animal models^25^. Cortisol would thus, via adrenergic stimulation, mobilize NEFA and glycogen, initiate the transport of FA for FAO (thus the release of mid-chain AC) and ultimately upregulate neonatal glucose levels, while oxytocin favours insulin and glucagon secretion for the postnatal glycemic regulation^22^. The lack of hormonal stimulations during PCS, and specifically of the elevated cortisol and oxytocin release, might cause the differences observed for glucose, NEFA, AC, and for c-peptide, respectively. The lack of hormonal stimulation, as well as differences in gluconeogenesis initiation, have been proposed as causal mechanisms linking PCS to long-term metabolic effects^24^. Unfortunately, we could not test these hypotheses due to lack of hormonal measurements in the study.

As caesarean section is a surgical procedure, parturient women receive a dose of anaesthesia, which can be general (mostly via thiopental or, more recently, via propofol^29^) or regional (spinal or epidural, via bupivacaine^30^). Neonatal differences attributable to the anesthesia are usually transient and not clinically relevant^31^. However, as these drugs can theoretically cross the placenta^29,31^, a confounding effect of the anaesthesia on the metabolome should be considered. For example, Irestedt and colleagues reported that, in babies delivered by PCS, catecholamines were higher in the epidural group than in the general anaesthesia group – albeit in both cases values were in several orders of magnitude lower than in babies delivered vaginally^32^. We did not have individual data about mode of anaesthesia in our collective, but it is general practice of the National Maternity Hospital to perform PCS under spinal anaesthesia. In line to that, a survey conducted in the United States in 2001 showed that “general anesthesia was still used in 15–30% of urgent-emergent cesarean deliveries in 2001. However, in elective cesarean deliveries, general anesthesia was used in less than 5% of cases in all sizes of hospitals”^33^. Thus, given the small sample size in our population (13 caesarean deliveries), we can assume that most to all PCS in our study were performed under local anaesthesia. Therefore, we conclude that our results about PCS are valid under spinal local anesthesia, but there might be small differences in case of general or other types of anaesthesia.

In our data, we see that both AC 18:2 and the marker of CPT1 activity involving 18:2 are lower in the cord blood of PCS rather than SVD babies. This marked difference involving 18:2 needs attention. While with our LC-MS approach we could not distinguish the position of the double bonds, most of the concentration of NEFA 18:2, and thus AC 18:2, can be attributed to linoleic acid, an omega-6 essential fatty acid (EFA). An impaired placental transport of long-chain EFA is known to occur in complicated pregnancies (e.g. gestational diabetes mellitus or intrauterine growth restriction)^34^, but our population did not present such cases. A possible explanation for this large difference might be due not so much to the PCS in itself, but rather to the usage of NEFA in SVD. A kinetic analysis in rat liver has shown that, when several NEFA species are available, CPT1 preferentially uses linoleic acid over other species, and especially over stearic acid (18:0, n-9), for turnover into its acylester^35^. Thus, in SVD babies, 18:2 would be the first NEFA to be metabolized into AC, and this would translate into much lower values of AC 18:2 and 18:2-CPT1 marker in PCS.

In the published literature, also the CB levels of alanine and of the branched chain amino acids (BCAA) isoleucine (Ile), leucine (Leu) and valine have been reported to be significantly lower in PCS than in SVD^36^. Alanine, in particular, is a preferred substrate for neonatal gluconeogenesis^23^. Our data only show weak trends for lower Ile, Val, ornithine and phenylalanine in PCS than in SVD. This might be explained by considering that our population had an elevated birthweight (around 4000 g), which might be associated with increased lipid depots and thus the preferred usage of NEFA rather than AA as substrates for gluconeogenesis. Phenylalanine, moreover, can be converted into tyrosine, which is a precursor of catecholamines; it is possible that the beginning of labour induces phenylalanine secretion in the fetal circulation for catecholamine production, whereas this does not happen in PCS. Tyrosine itself was not significantly associated with mode of delivery in our analysis, however it was lower in the PCS than in the SVD group.

### Anthropometry

As for metabolic markers, we observed no differences in growth and anthropometry up to 5 years of age between children born by SVD and IOL.

Babies born by PCS had trends for transiently higher weight (and length) z-scores (at 2 years) but these differences were not significant after correction for multiple testing and did not persist at 5 years. PCS was mostly performed around the 277^th^ gestation day (median), while SVD occurred at the 282^nd^ day, with no significant differences in the birthweights. Since elevated fetal weight increases the chance of PCS, it is very plausible that enhanced fetal growth, persisting also for some time after birth, might be the reason underlying both the elevated infant anthropometry and the earlier gestational age at birth. Unfortunately, the lack of steroid and hormonal measurements prevented us from testing this hypothesis.

The available literature about PCS and anthropometry or body mass identifies no consistent differences in infancy and childhood, but agrees on a higher risk for overweight in adult age for individuals born by PCS^24^. Our results suggest that mode of delivery does not have a major impact on early growth, but we observed minor differences at 2 years. We conjecture that these differences would disappear at 5 years due to lifestyle factors such as the child’s physical activity and food preferences^37,38^, but might reappear later in life, especially during or after puberty.

### Strengths and limitations

We have studied the impact of caesarean section and, for the first time, induction of labour, on the cord blood metabolome of 109 mother/child pairs. The mothers in the study had previously given birth to a macrosomic child and the studied population was at risk for fetal metabolic dysregulations. As pointed out by the WHO, more studies are needed to clarify the implications of IOL with mothers with metabolic dysregulations.

A major limitation of our study is the lack of detailed information about the delivery, especially about the type of anaesthesia for caesarean section (general vs. local, anesthetics), the type of induction and the duration of labour (possibly broken down in each stage); these pieces of information need to be collected and included in future studies tailored to investigate mode of delivery and metabolome. Another limitation of this study was the lack of steroid and hormonal measurements, which prevented us from further investigating our hypotheses regarding the interplay of fetal growth hormones, fetal stress at parturition, and cord metabolome. Finally, the small sample sizes of PCS and IOL vs. SVD might have prevented the identification of more subtle differences in the metabolites. However, our study can be used as a starting point for future comparisons, since the metabolomic research about mode of delivery has been so far scarce (for PCS) or non-existing (for IOL).

## Conclusion

In this cohort of secundigravid women with a previous macrosomic child, we have shown that IOL is not associated with differences in neonatal metabolism when compared to SVD, except for slightly elevated CPT1 activity markers, possibly precursor of postnatal gluconeogenesis. Elective PCS, on the contrary, induced profound changes in metabolites involved in fatty acid oxidation, glucose and c-peptide neonatal levels, possibly due to the lack in adrenergic stimulation and cortisol release. These data might contribute to future research on the impact of mode of delivery on short- and long-term metabolic outcomes, which could potentially have important ramifications for child health.

## Supplementary information

Supplement 1

Supplemental table 1
