# Supplementary material for: Caesarean section, but not induction of labour, is associated with major changes in cord blood metabolome
Source: Sci Rep. 2019 Nov 26;9:17562. doi: 10.1038/s41598-019-53810-1 (PMC6879512; doi:10.1038/s41598-019-53810-1)
Supplement: Supplementary file 1 — Supplement 1 [file 41598_2019_53810_MOESM1_ESM.docx]

Supplemental material

Caesarean section, but not induction of labour, is associated with major changes in cord blood metabolome

Linda Marchioro, Engy Shokry, Aisling A. Geraghty, Eileen C. O’Brien, Olaf Uhl, Berthold Koletzko, Fionnuala M. McAuliffe

**Methods for metabolomics analysis of amino acids, non-esterified fatty acids, acylcarnitines, tricarboxylic acid cycle intermediates and phospholipids.**

Proteins of 50 µL plasma were precipitated by adding 450 µL methanol including internal standards. After centrifugation the supernatant was split into aliquots for the analyses of individual methods detecting polar lipids, acylcarnitines, amino acids, non-esterified fatty acids and intermediates of the tricarboxylic acid cycle by liquid chromatography mass spectrometry (LC-MS/MS) analyses with multiple reaction monitoring mode.

*Amino acids.* A 50 µL aliquot of the supernatant was used for derivatization according to Harder et al.^1^. A set of labeled amino acid standards (set A, Cambridge Isotope Laboratories) mixed with L-Asparagine (15N2, 98%, Cambridge Isotope Laboratories) and L-Tryptophan (Indole-D5, 98%, Cambridge Isotope Laboratories) was used as internal standards. Amino acids were prepared by derivatization to butylester, and chromatographic separation was achieved with HPLC (1100, Agilent, Waldbronn, Germany) equipped with 150 x 2.1 mm, 3.5 µm particle size C18 HPLC column (X-Bridge, Waters, Milford, USA) and 0.1% heptafluoric butyric acid as ion pair reagent in both mobile phases A (water) and B (methanol). Mass spectrometry detection was performed with a triple quadrupole mass spectrometer (API2000, Sciex, Darmstadt, Germany) with atmospheric pressure chemical ionization source operating in positive ion ionization mode.

*Non-esterified fatty acids (NEFA)* An aliquot of 50 µl of the supernatant was analyzed using the method described by Hellmuth et al.^2^. Uniformly 13C-labeled palmitic acid was used as internal standard and 10 µl of the supernatant were injected to an HPLC system (1200, Agilent, Waldbronn, Germany) with a UPLC diphenyl column (Pursuit UPS Diphenyl, Agilent, Waldbronn, Germany) with 5 mM ammonium acetate and 2.1 mM acetic acid in water as mobile phase A and acetonitrile/isopropanol (80:20) as mobile phase B. An eluent flow rate of 700 mL/min was used to achieve chromatographic separation. A hybrid triple quadrupole mass spectrometer (4000 QTRAP, Sciex, Darmstadt, Germany) operating in negative electrospray ionization (ESI) multiple reaction monitoring (MRM) mode was used for MS detection. The analytical process was post-processed using Analyst software version 1.6.2.

*Acylcarnitines.* Acylcarnitines were measured via direct infusion without chromatographic separation as described in Uhl et al.^3^. D3-carnitine-C2, D3-carnitine-C8 and D3-carnitine-C16 (all Cambridge Isotope Laboratories, Tewksbury, MA, USA) were used as internal standards. Flow-injection analysis with isocratic elution with 76% isopropanol, 19% methanol and 5% water was used to measure acylcarnitines. The mass spectrometer (4000 QTRAP, Sciex, Darmstadt, Germany) was equipped with ESI and operated in positive ionization mode.

*Ketoacids and TCA intermediates*. Metabolites of the TCA cycle and ketoacids were measured by a modified method based on previously published procedures^4,5^. D3-methylmalonic acid (Cambridge Isotope Laboratories, Tewksbury, MA, USA) was used as internal standard. 100 μL of the supernatant were evaporated to dryness and re-suspended in 50 μL water. 5 μL of the extracted samples were injected by HPLC system (1200, Agilent, Waldbronn, Germany) on a Kinetex F5 core-shell HPLC column, 150 x 2.1 mm, 2.6 μm particle size (Phenomenex, Aschaffenburg, Germany) for separation of molecular species. The mobile phase A was water with 1% formic acid and mobile phase B was composed of methanol/ isopropanol (50:50) with 1% formic acid. The gradient elution at a flow rate of 250 μL/min was held constant for 1 minute with 1% B, raised to 65% B within 6 minutes, and turned back to initial conditions of 1%B within 0.5 minutes. The triple quadrupole mass spectrometer (4000QTRAP, Sciex, Darmstadt, Germany) was operated in negative scheduled multiple reaction monitoring mode using ESI.

*Phospholipids.* Phospholipids were analyzed via flow-injection mass spectrometry (FIA-MS/MS) as described in Uhl et al.^3^ using Lyso-PC(13:0) and PC(14:0/14:0) (Avanti Polar Lipids, Alabaster, Alabama, USA) as internal standards. 30 μL of the centrifuged supernatant were mixed for 20 min at 600 rpm with 500 µl methanol containing 1.2 mM ammonium acetate and injected in a triple quadrupole mass spectrometer (QTRAP4000, Sciex, Darmstadt, Germany) coupled to a LC system (1200 Agilent, Waldbronn, Germany). ESI was used in positive ionization mode. MS/MS analysis was run in MRM with 184 Da (choline head group) as product ion for the PL. Analyst 1.6.2 software, followed by in-house processing with R^6^, was used for post-processing.

**References**

1 Harder, U., Koletzko, B. & Peissner, W. Quantification of 22 plasma amino acids combining derivatization and ion-pair LC-MS/MS. *Journal of chromatography. B, Analytical technologies in the biomedical and life sciences* **879**, 495-504, doi:10.1016/j.jchromb.2011.01.010 (2011).

2 Hellmuth, C., Weber, M., Koletzko, B. & Peissner, W. Nonesterified fatty acid determination for functional lipidomics: comprehensive ultrahigh performance liquid chromatography-tandem mass spectrometry quantitation, qualification, and parameter prediction. *Analytical chemistry* **84**, 1483-1490, doi:10.1021/ac202602u (2012).

3 Uhl, O., Fleddermann, M., Hellmuth, C., Demmelmair, H. & Koletzko, B. Phospholipid Species in Newborn and 4 Month Old Infants after Consumption of Different Formulas or Breast Milk. *PloS one* **11**, e0162040, doi:10.1371/journal.pone.0162040 (2016).

4 Luo, B., Groenke, K., Takors, R., Wandrey, C. & Oldiges, M. Simultaneous determination of multiple intracellular metabolites in glycolysis, pentose phosphate pathway and tricarboxylic acid cycle by liquid chromatography-mass spectrometry. *Journal of chromatography. A* **1147**, 153-164, doi:10.1016/j.chroma.2007.02.034 (2007).

5 Birkler, R. I. *et al.* A UPLC-MS/MS application for profiling of intermediary energy metabolites in microdialysis samples--a method for high-throughput. *Journal of pharmaceutical and biomedical analysis* **53**, 983-990, doi:10.1016/j.jpba.2010.06.005 (2010).

6 R: A language and environment for statistical computing (R Foundation for Statistical Computing, Vienne, Austria, 2018).
